# Voices change my name

**DOI:** 10.1192/j.eurpsy.2022.2070

**Published:** 2022-09-01

**Authors:** C. Vallecillo Adame, T. Jiménez Aparicio, C. De Andrés Lobo, A. Gonzaga Ramírez, M. Queipo De Llano De La Viuda, G. Guerra Valera, I. Santos Carrasco, J. Gonçalves Cerejeira, B. Rodríguez Rodríguez, M. Fernández Lozano, M.J. Mateos Sexmero, N. Navarro Barriga, N. De Uribe Viloria, G. Medina Ojeda, L. Rodriguez Andrés

**Affiliations:** 1 Hospital Clínico Universitario, Psiquiatría, Valladolid, Spain; 2 Hospital Clínico Universitario de Valladolid, Psiquiatría, VALLADOLID, Spain; 3 Hospital Clínico Universitario de Valladolid, Psychiatry, Valladolid, Spain; 4 Hospital Universitario Fundación de Alcorcón, Psychiatry, Madrid, Spain; 5 Sacyl, Hospital Clínico Universitario Valladolid, Psiquiatría, Valladolid, Spain; 6 Hospital Clinico Universitario de Valladolid, Psychiatry, Valladolid, Spain

**Keywords:** Gender Dysphoria, schizophrénia, Transgender, Psychosis

## Abstract

**Introduction:**

We present the clinical case of a patient where the psychotic clinic coexists with gender dysphoria. This scenario can be the result of a change in gender identity derived from the psychotic process or appear independently of it.

**Objectives:**

We want to explain the importance of knowing how to act with a patient in whom these two processes coexist.

**Methods:**

20-year-old woman, with no history of mental health. She comes to the emergency department for behavioral alteration. The family observes strange behaviors, unmotivated laughter, soliloquies and aggressive episodes. Abandonment of studies, hobbies and radical physical change. Delusions of prejudice and self-referential delusions. Possible phenomena of echo and diffusion of the thought. Auditory hallucinations talking to her in male gender, since then she presents doubts about her sexual identity and manifests her desire to change sex. Altered judgment of reality.

**Results:**

During admission, we started treatment with an antipsychotic with good tolerance and she was referred to mental health team, where psychopharmacological treatment was adjusted with good response. In the following medical appointments the psychotic clinic disappeared at the same time that sexual identification was completely restored and made a critique of the behavior and experiences.

**Conclusions:**

This case highlights the importance of assessing the chronology of symptoms, the patient’s criticality, the response to antipsychotic treatment and the need to exclude the psychotic background of the desire for gender reassignment before making a therapeutic decision.

**Disclosure:**

No significant relationships.

